# Predicting drug target interactions using meta-path-based semantic network analysis

**DOI:** 10.1186/s12859-016-1005-x

**Published:** 2016-04-12

**Authors:** Gang Fu, Ying Ding, Abhik Seal, Bin Chen, Yizhou Sun, Evan Bolton

**Affiliations:** National Center for Biotechnology Information, National Library of Medicine, National Institutes of Health, 8600 Rockville Pike, Bethesda, MD USA; School of Informatics & Computing, Indiana University, 107 S. Indiana Ave, Bloomington, IN USA; School of Information Management, Wuhan University, Wuchang, Wuhan, Hubei China; Department of Medicine, Stanford University, 450 Serra Mall, Stanford, CA USA; College of Computer and Information Science, Northeastern University, 360 Huntington Avenue, Boston, MA USA

**Keywords:** Semantic network analysis, Link prediction, Meta-path topological feature, Machine learning, Random forest

## Abstract

**Background:**

In the context of drug discovery, drug target interactions (DTIs) can be predicted based on observed topological features of a semantic network across the chemical and biological space. In a semantic network, the types of the nodes and links are different. In order to take into account the heterogeneity of the semantic network, meta-path-based topological patterns were investigated for link prediction.

**Results:**

Supervised machine learning models were constructed based on meta-path topological features of an enriched semantic network, which was derived from Chem2Bio2RDF, and was expanded by adding compound and protein similarity neighboring links obtained from the PubChem databases. The additional semantic links significantly improved the predictive performance of the supervised learning models. The binary classification model built upon the enriched feature space using the Random Forest algorithm significantly outperformed an existing semantic link prediction algorithm, Semantic Link Association Prediction (SLAP), to predict unknown links between compounds and protein targets in an evolving network. In addition to link prediction, Random Forest also has an intrinsic feature ranking algorithm, which can be used to select the important topological features that contribute to link prediction.

**Conclusions:**

The proposed framework has been demonstrated as a powerful alternative to SLAP in order to predict DTIs using the semantic network that integrates chemical, pharmacological, genomic, biological, functional, and biomedical information into a unified framework. It offers the flexibility to enrich the feature space by using different normalization processes on the topological features, and it can perform model construction and feature selection at the same time.

**Electronic supplementary material:**

The online version of this article (doi:10.1186/s12859-016-1005-x) contains supplementary material, which is available to authorized users.

## Background

Chemogenomics [1, 2] and chemical systems biology [3, 4] aim to accelerate drug discovery inexpensively through *in silico* predictions, based on a network with enriched drug-target-disease relationships [5]. Integrated chemical and biological networks can be used to hypothesize new clinical indications for approved drugs with desired safety profiles, and to propose new combination therapy design [6, 7]. Drug-target interaction networks can also be utilized to interpret clinical side effects by revealing modes of drug actions [8]. Semantic standards and technologies facilitate seamless data integration across multiple domains, and enable the construction of a heterogeneous network consisting of various biological entities of different types, such as compounds, proteins, and genes [9]. Several semantically linked datasets, such as PubChemRDF [10], Chem2Bio2Rdf [11], Bio2RDF [12], Open PHACTS [13], and ChEMBL RDF [14], have been published to promote large-scale data mining in drug discovery. A statistical model, called Semantic Link Association Prediction (SLAP), has been applied to Chem2Bio2RDF to predict direct links between compounds and proteins based on their indirect links or paths with other biological objects, such as substructures, diseases, side effects, and pathways [15]. It has been demonstrated that SLAPas a novel and validated approach to predict drug-target interactions (DTIs) outperformed existing alternatives.

Predicting DTI is equivalent to link prediction, which is a fundamental problem and long-standing challenge in complex network analysis [16]. In social networks, topological proximity, measured based on observed network data, can be used to suggest future interactions between individuals [17]. In the context of drug discovery, biological networks can be similarly leveraged to identify potential associations between compounds and protein targets. Typical network-based DTI predictions are often based on similarity profiles calculated from common neighbors or direct connections, and are usually limited to bipartite networks [18–21]. However, most similarity-based link prediction algorithms designed for homogeneous networks cannot take into account the heterogeneous types and relations defined in semantic networks; furthermore, it is fairly challenging to consider the long paths connecting two end nodes (indirect connections), which can significantly increase large volumes of randomness in the connectivity. Therefore, we incorporated meta-path topological features [22] for link prediction. A meta-path is a composite relation, denoting a sequence of adjacent links between any two objects in a heterogeneous network. Adjacent links are defined with distinct semantics, so different combinations of adjacent links in sequences contribute distinguishably for link prediction. It has been proven that meta-path-based similarity can improve the performance of information retrieval in heterogeneous information networks [23].

A meta-path defines a certain type of paths linking the starting and ending objects. The total number of paths belonging to a specific meta-path is animportant topological feature to evaluate the strength of associations between starting and ending objects, which is often called path count. For instance, a compound and a protein target can be connected through multiple paths of different types: (A) compound $\overset{similar\ to}{\to }$ compound $\overset{binds\ to}{\to }$ protein; (B) compound $\overset{binds\ to}{\to }$ protein $\overset{binds\ to}{\to }$ compound $\overset{binds\ to}{\to }$ protein; and (C) compound $\overset{has\  part}{\to }$ substructure $\overset{part\  of}{\to }$ compound $\overset{binds\ to}{\to }$ protein $\overset{similar\ to}{\to }$ protein. Three meta-paths connect the starting compound to the ending protein: meta-path (A) indicates that the compound most likely binds to a protein to which another structurally similar compound binds; meta-path (B) shows that two compounds sharing an observed protein target may share another protein target as well; meta-path (C) specifies that two compounds sharing a common substructure may bind to two different protein targets that have similar protein sequences. SLAP employs a statistical model to evaluate the importance of each meta-path in link prediction, which is evaluated individually based on the distribution of its connectivity property over a set of randomly sampled drug-target pairs. Several meta-paths are selected according to their statistical significances, and the aggregated connectivity properties of the selected meta-paths are used to predict DTI.

The present work provides an alternative DTI approach to SLAP. Rather than using a statistical model to study the significance of meta-path topological features, we propose a framework to take advantage of machine learning algorithms, including Random Forest (RF) and Support Vector Machine (SVM), to construct binary classification models to predict DTI. A more complete drug-target connectivity map can be constructed using the predicted links. By using machine learning models, feature importance (i.e., the contributions of different meta-paths to the link prediction) can be calculated at the same time as the classification models are built. Additionally, SLAP only considers path counts as a topological feature; whereas our approach can apply different kinds of normalization processes to path counts, including random walk, normalized path count, and symmetric random walk [23] to further enrich the topological feature space. In order to compare our approach with SLAP, we have carried out link prediction experiments on a semantic network, called Chem2Bio2Rdf, which focuses on drug candidates and their biological annotations. Although the proposed approach was just used to construct a more complete drug-target connectivity map in the present study, it can be generalized as a framework to leverage machine learning algorithms to study the topological features of the heterogeneous network for link prediction. Structural similarity links between compounds and sequence similarity links between proteins were added to expand the semantic network. The usefulness of similarity neighboring links from PubChem resources [24] is examined in the context of semantic link prediction.

## Methods

### Semantic network

In the Chem2Bio2RDF semantic network, nine distinct semantic types are presented, including compounds, proteins, adverse side effects, Gene Ontology (GO) annotations, ChEBI types, substructures, tissues, biological pathways, and diseases; ten different semantic links are incorporated, including links from compounds to ChEBI types, from compounds to proteins, from compounds to substructures, from adverse side effects to compounds, from diseases to compounds, from proteins to proteins (referring to protein-protein interactions), from proteins to GO annotations, from diseases to proteins, from pathways to proteins, and from tissues to proteins. In order to enhance link prediction performance, we enriched the linked dataset by adding two more semantic links: compound neighboring links based on 2D structural similarity, and protein neighboring links, based on sequence similarity. The similarity neighboring links were obtained from PubChem databases [25, 26]. A total of twelve adjacency matrixes were computed based on the semantic links between any two objects. The elements of the adjacency matrixes have two values: ‘0,’ indicating unobserved links, and ‘1,’ indicating observed links. The semantics and statistics of adjacency matrixes were enumerated in Table 1; these were used to calculate the meta-path-based topological features. It is noteworthy that all the semantic links in the Chem2Bio2RDF dataset are reversible, and the adjacency matrix for the reverse semantic links can be obtained through a transpose of the original adjacency matrix.

**Table 1:** The semantics and statistics of adjacency matrixes

Index	Semantics	From	Number of Rows	To	Number of Colums	Count^*a*^
A1	has ChEBI type	compound	258030	ChEBI type	2777	14633
A2	binds to	compound	258030	protein	22056	528831
A3	has part	compound	258030	substructure	290	6127
A4	induced by	adverse side effect	1051	compound	258030	9004
A5	treated by	disease	1284	compound	258030	927
A6	interacts with	protein	22056	protein	22056	72773
A7	has GO annotation	protein	22056	GO annotation	9710	89688
A8	caused by	disease	1284	protein	22056	2676
A9	has participants	pathway	192	protein	22056	10796
A10	expresses	tissue	507	protein	22056	9905
A11	similar to	compound	258030	compound	258030	6184722
A12	similar to	protein	22056	protein	22056	261158

^*a*^The number of non-zero elements in adjacency matrix

### Meta-path-based topological features

**Fig. 1:**
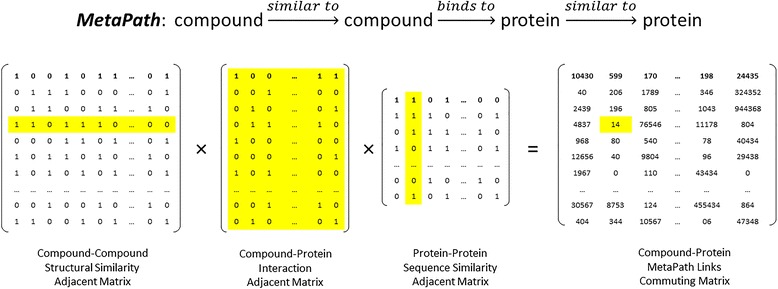
Schematic representation of calculations of commuting matrix C15 through multiplying A2, A11, and A12

The meta-path topological features were encoded in commuting matrixes, calculated by multiplying several adjacency matrixes. To predict the links from compounds to proteins, we exhaustively enumerate all the possible meta-paths, yielding a total of 51 meta-paths. Each commuting matrix represents a certain type of meta-path of a given length. The length of the meta-paths equals the number of multiplied adjacency matrixes. Out of 51 commuting matrixes, 4 meta-paths are of length 2; 11 meta-paths are of length 3; and 36 meta-paths are of length 4. The meta-paths with length greater than 4 are considered to be too long to make a significant contribution to link prediction. The elements in the commuting matrix indicate the number of path instances linking compounds to proteins, and have non-negative integer values. The semantics and statistics of commuting matrixes were enumerated in Table 2. For instance, the commuting matrix C15 represents a meta-path: compound $\overset{similar\ to}{\to }$ compound $\overset{binds\ to}{\to }$ protein $\overset{similar\ to}{\to }$ protein, which was calculated by multiplying three adjacency matrixes: A2, A11, and A12 (Fig. 1). All of the matrix multiplications were carried out using the Armadillo C++ linear algebra library [27], and all of the adjacency and commuting matrixes were encoded as sparse matrixes to reduce memory consumption.

**Table 2:** The semantics and statistics of commuting matrixes

Index	Semantics	Count^*a*^	Max^*b*^
C1	$\mathrm{compound}\ \overset{similar\;to}{\to}\;\mathrm{compound}\overset{binds\;to}{\to}\mathrm{protein}$	1995778	395
C2	$\mathrm{compound}\ \overset{binds\;to}{\to}\;\mathrm{protein}\overset{interacts\; with}{\to}\mathrm{protein}$	4878633	20
C3	$\mathrm{compound}\ \overset{binds\;to}{\to}\;\mathrm{protein}\overset{similar\ to}{\to}\mathrm{protein}$	30665527	84
C4	$\mathrm{compound}\ \overset{treats}{\to}\;\mathrm{disease}\overset{caused\;by}{\to}\mathrm{protein}$	6178	3
C5	$\mathrm{compound}\ \overset{similar\;to}{\to}\;\mathrm{compound}\overset{binds\;to}{\to}\mathrm{protein}\overset{interacts\ with}{\to}\mathrm{protein}$	15086309	934
C6	$\mathrm{compound}\ \overset{similar\;to}{\to}\;\mathrm{compound}\overset{binds\;to}{\to}\mathrm{protein}\overset{similar\ to}{\to}\mathrm{protein}$	49226573	1163
C7	$\mathrm{compound}\ \overset{binds\;to}{\to}\;\mathrm{protein}\overset{binds\;to}{\to}\mathrm{compound}\overset{binds\ to}{\to}\mathrm{protein}$	126339670	30400
C8	$\mathrm{compound}\ \overset{has\; part}{\to}\;\mathrm{substructure}\overset{part\; of}{\to}\mathrm{compound}\overset{binds\ to}{\to}\mathrm{protein}$	922056	202
C9	$\mathrm{compound}\ \overset{has\; type}{\to}\;\mathrm{ChEBI}\ \mathrm{type}\overset{type\ of}{\to}\mathrm{compound}\overset{binds\ to}{\to}\mathrm{protein}$	709802	324
C10	$\mathrm{compound}\ \overset{induces}{\to}\;\mathrm{adverse}\ \mathrm{side}\ \mathrm{effect}\overset{induced\kern0.5em by}{\to}\mathrm{compound}\overset{binds\ to}{\to}\mathrm{protein}$	420616	194
C11	$\mathrm{compound}\ \overset{treats}{\to}\;\mathrm{disease}\overset{treated\;by}{\to}\mathrm{compound}\overset{binds\ to}{\to}\mathrm{protein}$	68479	25
C12	$\mathrm{compound}\ \overset{binds\;to}{\to}\;\mathrm{protein}\overset{has\; annotation}{\to}\mathrm{GO}\ \mathrm{annotation}\overset{annotation\ of}{\to}\mathrm{protein}$	316095950	335
C13	$\mathrm{compound}\ \overset{binds\;to}{\to}\;\mathrm{protein}\overset{participates\; in}{\to}\mathrm{pathway}\overset{has\ participants}{\to}\mathrm{protein}$	82834409	328
C14	$\mathrm{compound}\ \overset{binds\;to}{\to}\;\mathrm{protein}\overset{expressed\; in}{\to}\mathrm{tissue}\overset{expresses}{\to}\mathrm{protein}$	53586080	76
C15	$\mathrm{compound}\ \overset{binds\;to}{\to}\;\mathrm{protein}\overset{causes}{\to}\mathrm{disease}\overset{caused\ by}{\to}\mathrm{protein}$	1360337	10
C16	$\mathrm{compound}\ \overset{binds\;to}{\to}\;\mathrm{protein}\overset{binds\;to}{\to}\mathrm{compound}\overset{binds\ to}{\to}\mathrm{protein}\overset{interact\ with}{\to}\mathrm{protein}$	522513250	142290
C17	$\mathrm{compound}\ \overset{binds\;to}{\to}\;\mathrm{protein}\overset{binds\;to}{\to}\mathrm{compound}\overset{treats}{\to}\mathrm{disease}\overset{caused\ by}{\to}\mathrm{protein}$	12963831	498
C18	$\mathrm{compound}\ \overset{binds\;to}{\to}\mathrm{protein}\ \overset{binds\;to}{\to}\mathrm{compound}\ \overset{similar\;to}{\to}\mathrm{compound}\ \overset{binds\;to}{\to}\mathrm{protein}$	201052081	777576
C19	$\mathrm{compound}\ \overset{binds\;to}{\to}\mathrm{protein}\ \overset{binds\;to}{\to}\mathrm{compound}\ \overset{binds\;to}{\to}\mathrm{protein}\ \overset{similar\;to}{\to}\mathrm{protein}$	356122463	445332
C20	$\mathrm{compound}\ \overset{type\; of}{\to}\mathrm{ChEBI}\ \mathrm{type}\ \overset{type\; of}{\to}\mathrm{compound}\ \overset{binds\;to}{\to}\mathrm{protein}\overset{interacts\ with}{\to}\mathrm{protein}$	2333739	2711
C21	$\mathrm{compound}\ \overset{type\; of}{\to}\mathrm{ChEBI}\ \mathrm{type}\ \overset{type\; of}{\to}\mathrm{compound}\overset{treats}{\to }\ \mathrm{disease}\overset{caused\;by}{\to}\mathrm{protein}$	190923	194
C22	$\mathrm{compound}\overset{type\; of}{\to}\mathrm{ChEBI}\ \mathrm{type}\overset{type\; of}{\to}\mathrm{compound}\ \overset{binds\ to}{\to}\mathrm{protein}\ \overset{similar\;to}{\to}\mathrm{protein}$	1463743	8639
C23	$\mathrm{compound}\overset{type\; of}{\to}\mathrm{ChEBI}\ \mathrm{type}\overset{type\ of}{\to}\mathrm{compound}\overset{similar\ to}{\to}\mathrm{compound}\overset{binds\ to}{\to}\mathrm{protein}$	922257	8402
C24	$\mathrm{compound}\overset{treats}{\to}\mathrm{disease}\overset{treated\ by}{\to}\mathrm{compound}\overset{binds\;to}{\to}\mathrm{protein}\overset{interacts\ with}{\to}\mathrm{protein}$	371971	162
C25	$\mathrm{compound}\overset{treats}{\to}\mathrm{disease}\overset{treated\ by}{\to}\mathrm{compound}\overset{treats}{\to}\mathrm{disease}\overset{caused\ by}{\to}\mathrm{protein}$	38708	91
C26	$\mathrm{compound}\overset{treats}{\to}\mathrm{disease}\overset{treated\ by}{\to}\mathrm{compound}\overset{binds\;to}{\to}\mathrm{protein}\overset{similar\;to}{\to}\mathrm{protein}$	493976	400
C27	$\mathrm{compound}\overset{treats}{\to}\mathrm{disease}\overset{treated\ by}{\to}\mathrm{compound}\overset{similar\;to}{\to}\mathrm{compound}\overset{binds\;to}{\to}\mathrm{protein}$	106013	710
C28	$\mathrm{compound}\overset{induces}{\to}\mathrm{adverse}\ \mathrm{side}\ \mathrm{effect}\overset{induced\ by}{\to}\mathrm{compound}\overset{binds\;to}{\to}\mathrm{protein}\overset{interacts\ with}{\to}\mathrm{protein}$	1766464	1622
C29	$\mathrm{compound}\overset{induces}{\to}\mathrm{adverse}\ \mathrm{side}\ \mathrm{effect}\overset{induced\ by}{\to}\mathrm{compound}\ \overset{treats}{\to}\mathrm{disease}\overset{caused\ by}{\to}\mathrm{protein}$	168841	106
C30	$\mathrm{compound}\overset{induces}{\to}\mathrm{adverse}\ \mathrm{side}\ \mathrm{effect}\overset{induced\ by}{\to}\mathrm{compound}\overset{binds\;to}{\to}\mathrm{protein}\overset{similar\;to}{\to}\mathrm{protein}$	1193429	5571
C31	$\mathrm{compound}\overset{induces}{\to}\mathrm{adverse}\ \mathrm{side}\ \mathrm{effect}\overset{induced\ by}{\to}\mathrm{compound}\overset{similar\;to}{\to}\mathrm{compound}\overset{binds\;to}{\to}\mathrm{protein}$	765725	2744
C32	$\mathrm{compound}\overset{has\ part}{\to}\mathrm{substructure}\overset{part\ of}{\to}\mathrm{compound}\overset{binds\;to}{\to}\mathrm{protein}\overset{interacts\ with}{\to}\mathrm{protein}$	3465967	902
C33	$\mathrm{compound}\overset{has\ part}{\to}\mathrm{substructure}\overset{part\ of}{\to}\mathrm{compound}\overset{treats}{\to}\mathrm{disease}\overset{caused\ by}{\to}\mathrm{protein}$	355993	96
C34	$\mathrm{compound}\overset{has\ part}{\to}\mathrm{substructure}\overset{part\ of}{\to}\mathrm{compound}\overset{binds\;to}{\to}\mathrm{protein}\overset{similar\;to}{\to}\mathrm{protein}$	2175094	2753
C35	$\mathrm{compound}\overset{has\ part}{\to}\mathrm{substructure}\overset{part\ of}{\to}\mathrm{compound}\overset{similar\;to}{\to}\mathrm{compound}\overset{binds\;to}{\to}\mathrm{protein}$	1206786	12048
C36	$\mathrm{compound}\overset{binds\;to}{\to}\mathrm{protein}\overset{interacts\ with}{\to}\mathrm{protein}\ \overset{has\ annotation}{\to}\mathrm{GO}\ \mathrm{annotation}\overset{annotation\ of}{\to}\mathrm{protein}$	1064451402	1929
C37	$\mathrm{compound}\overset{treats}{\to}\mathrm{disease}\overset{caused\ by}{\to}\mathrm{protein}\overset{has\ annotation}{\to}\mathrm{GO}\ \mathrm{annotation}\overset{annotation\ of}{\to}\mathrm{protein}$	2280505	136
C38	$\mathrm{compound}\overset{binds\;to}{\to}\mathrm{protein}\overset{similar\;to}{\to}\mathrm{protein}\overset{has\ annotation}{\to}\mathrm{GO}\ \mathrm{annotation}\overset{annotation\ of}{\to}\mathrm{protein}$	1480055439	50667
C39	$\mathrm{compound}\overset{similar\ to}{\to}\mathrm{compound}\overset{binds\;to}{\to}\mathrm{protein}\overset{has\ annotation}{\to}\mathrm{GO}\ \mathrm{annotation}\overset{annotation\ of}{\to}\mathrm{protein}$	582316693	7765
C40	$\mathrm{compound}\overset{binds\;to}{\to}\mathrm{protein}\overset{interacts\ with}{\to}\mathrm{protein}\overset{participates\ in}{\to}\mathrm{pathway}\overset{has\kern0.5em participants}{\to}\mathrm{protein}$	246398750	2989
C41	$\mathrm{compound}\overset{treats}{\to}\mathrm{disease}\overset{caused\ by}{\to}\mathrm{protein}\overset{participates\ in}{\to}\mathrm{pathway}\overset{has\ participants}{\to}\mathrm{protein}$	486267	183
C42	$\mathrm{compound}\overset{binds\;to}{\to}\mathrm{protein}\overset{similar\;to}{\to}\mathrm{protein}\overset{\ participates\ in}{\to}\mathrm{pathway}\overset{\ has\kern0.5em participants}{\to}\mathrm{protein}$	358346529	73327
C43	$\mathrm{compound}\overset{similar\ to}{\to}\mathrm{compound}\overset{binds\;to}{\to}\mathrm{protein}\overset{\ participates\kern0.5em in}{\to}\mathrm{pathway}\overset{\ has\kern0.5em participants}{\to}\mathrm{protein}$	149299008	7543
C44	$\mathrm{compound}\overset{binds\;to}{\to}\mathrm{protein}\overset{interacts\ with}{\to}\mathrm{protein}\overset{causes}{\to}\mathrm{disease}\ \overset{caused\kern0.5em by}{\to}\mathrm{protein}$	7603639	44
C45	$\mathrm{compound}\overset{treats}{\to}\mathrm{disease}\overset{caused\ by}{\to}\mathrm{protein}\overset{causes}{\to}\mathrm{disease}\overset{caused\ by}{\to}\mathrm{protein}$	27193	63
C46	$\mathrm{compound}\overset{binds\;to}{\to}\mathrm{protein}\overset{similar\;to}{\to}\mathrm{protein}\overset{causes}{\to}\mathrm{disease}\overset{caused\ by}{\to}\mathrm{protein}$	26747896	802
C47	$\mathrm{compound}\overset{similar\;to}{\to}\mathrm{compound}\overset{binds\;to}{\to}\mathrm{protein}\overset{causes}{\to}\mathrm{disease}\overset{caused\ by}{\to}\mathrm{protein}$	4159753	313
C48	$\mathrm{compound}\overset{binds\;to}{\to}\mathrm{protein}\overset{interacts\ with}{\to}\mathrm{protein}\overset{expressed\ in}{\to}\mathrm{tissue}\overset{expresses}{\to}\mathrm{protein}$	222288200	453
C49	$\mathrm{compound}\overset{treats}{\to}\mathrm{disease}\overset{caused\;by}{\to}\mathrm{protein}\overset{expressed\ in}{\to}\mathrm{tissue}\overset{expresses}{\to}\mathrm{protein}$	300620	27
C50	$\mathrm{compound}\overset{binds\ to}{\to}\mathrm{protein}\overset{similar\;to}{\to}\mathrm{protein}\overset{expressed\ in}{\to}\mathrm{tissue}\overset{expresses}{\to}\mathrm{protein}$	431134094	5974
C51	$\mathrm{compound}\overset{similar\;to}{\to}\mathrm{compound}\overset{binds\;to}{\to}\mathrm{protein}\overset{expressed\ in}{\to}\mathrm{tissue}\overset{expresses}{\to}\mathrm{protein}$	117576353	2031

^*a*^ The number of non-zero elements in commuting matrix; ^*b*^ the max value of element in commuting matrix.

Two measures of topological features were calculated. Path count (*PC*_*i*,*j*_) measures the number of path instances between nodes *i* and *j*, which corresponds to the value of element in the commuting matrix. We also applied Random Walk (RW) as a normalization process to the number of path instances, based on the overall connectivity of the network. RW was calculated as $$ \raisebox{1ex}{$P{C}_{i,j}$}\!\left/ \!\raisebox{-1ex}{$P{C}_{i,\bullet }$}\right. $$, where *PC*_*i*,•_ are row-wise summations.

### Machine learning dataset

In order to build supervised learning models, both positive and negative labels are required. We treated observed links between compounds and protein targets as positive labels. A total of 5,387 positively labeled links from Drugbank were collected, which were used to evaluate the predictive performance of the SLAP algorithm [15]. The unobserved links in the dataset can be either spurious links or potential future links. In order to obtain experimental evidence for the negative labels, we surveyed the PubChem BioAssay database [28]: if the experimental bioactivity value is greater than 10 μM, the link of a compound protein pair is negatively labeled. Accordingly, we obtained 26,682 negative labels out of over 5.6 billion unobserved links between compounds and proteins in the Chem2Bio2RDF semantic network. In order to assess predictive performance without prior knowledge, the positively labeled links were removed from Chem2Bio2RDF when the meta-path-based topological features were calculated. The positively and negatively labeled links were combined and randomly split into training and test sets by a ratio of 2:1. In the training set, there are 3,591 positively labeled links and 17,788 negatively labeled links. In the test set, there are 1,796 positively labeled links and 8,894 negatively labeled links.

The network evolves as new links are identified over time. In order to further examine the ability of the proposed framework to identify the evolution of network connectivity, a much larger set of DTIs were collected from the PubChem BioAssay database. PubChem BioAssay categorizes depositor-provided bioactivities between compounds and protein targets into active, inactive, and unspecified groups, according to assay descriptions and activity values. If the interactions between compounds and protein targets are categorized as active in PubChem BioAssay, and the active interaction pairs have reported activity values of less than 1 μM, the links are positively labeled; if the interactions between compounds and proteins are categorized as inactive in PubChem BioAssay, and there are reported activities for the interactions, the links are negatively labeled. A set of 145,622 positively labeled links contained in the current Chem2Bio2RDF semantic network, plus 600,000 negatively labeled links, constitute a training set; another set of 43,159 positively labeled links that are not contained in the current Chem2Bio2RDF semantic network, but are true positive DTIs, identified through bioassay experiments, plus195,000 negatively labeled links, comprise the test set. Since the positive DTIs in the test set were obtained after construction of the network, this independent test set is used to examine the ability to predict the links in the future network based on the topological features of the current network.

### Binary classification models

In order to demonstrate how well the similarity neighboring links obtained from PubChem databases can improve link prediction performance, we have constructed different machine learning models, based on two sets of path count topological features. Feature set ***I*** does not include any meta-paths involving similarity neighboring links, so it only contains 29 path count topological features. Feature set ***II*** includes all of the path counts encoded in 51 commuting matrixes. We also examined the improvement of predictive performance using an enriched topological feature space. RW normalization was applied to 51 path count topological features, and by combining the path counts and random walks, we obtained feature set ***III***, which contains 102 topological features.

Two popular machine learning algorithms were investigated. Random forest (RF) represents a collection of decision trees, which are grown from bootstrap samples of the training data without pruning, and make predictions based on majority votes of the ensemble trees [29]. RF takes advantage of Out-of-Bag (OOB) error as an unbiased estimate of generalized test error, so there is no need to run cross-validation. RF can calculate the importance of features as well. The values for a given feature are permuted across all of the compound-protein pairs. Either classification accuracies or node impurities (Gini indexes) are measured before and after permutations, and the difference in the measures is used to evaluate feature importance. A default value for the number of trees was used (*ntree* = 500) in the present study, which has been proven to be satisfactory in most cases [30]. The optimal value for tuning parameter *mtry* was identified by a grid search.

In contrast to the tree-based model, Support Vector Machine (SVM) is based on a statistical learning theory derived from the structural risk minimization principle and Vapnik-Chervonenkis (VC) dimension [31]. A soft margin SVM with radial basis function (RBF) kernel in the Gaussian form was used in the present study. The optimal values for tuning parameters (*C* and *λ*) were determined by a grid search using 10-fold cross-validation.

The classification performances were evaluated using the F_1_-score [32], which is the harmonic mean of precision and recall.

1 $$ {\mathrm{F}}_1\;\mathrm{score}:\;\frac{2TP}{2TP+FP+FN} $$

F_1_-score can be used for statistical hypothesis testing, in particular, for imbalanced datasets. Both RF and SVM can calculate the probabilities of classifications, and rankings can be derived from the probability calculations. The predictive performance on rankings was evaluated according to Receiver Operating Characteristic (ROC) and Precision Recall (PR) curves for all of the models. The area under the curve for ROC (AUCROC) and PR (AUCPR) were calculated using the natural spline interpolation encoded in the R package ‘Miscellaneous Esoteric Statistical Scripts’ (MESS). The early hit recognitions that are considered more important in virtual screening experiments were evaluated using Boltzmann-enhanced discrimination of ROC (BEDROC), which was calculated using the R package ‘enrichvs.’

## Results and discussion

**Fig. 2:**
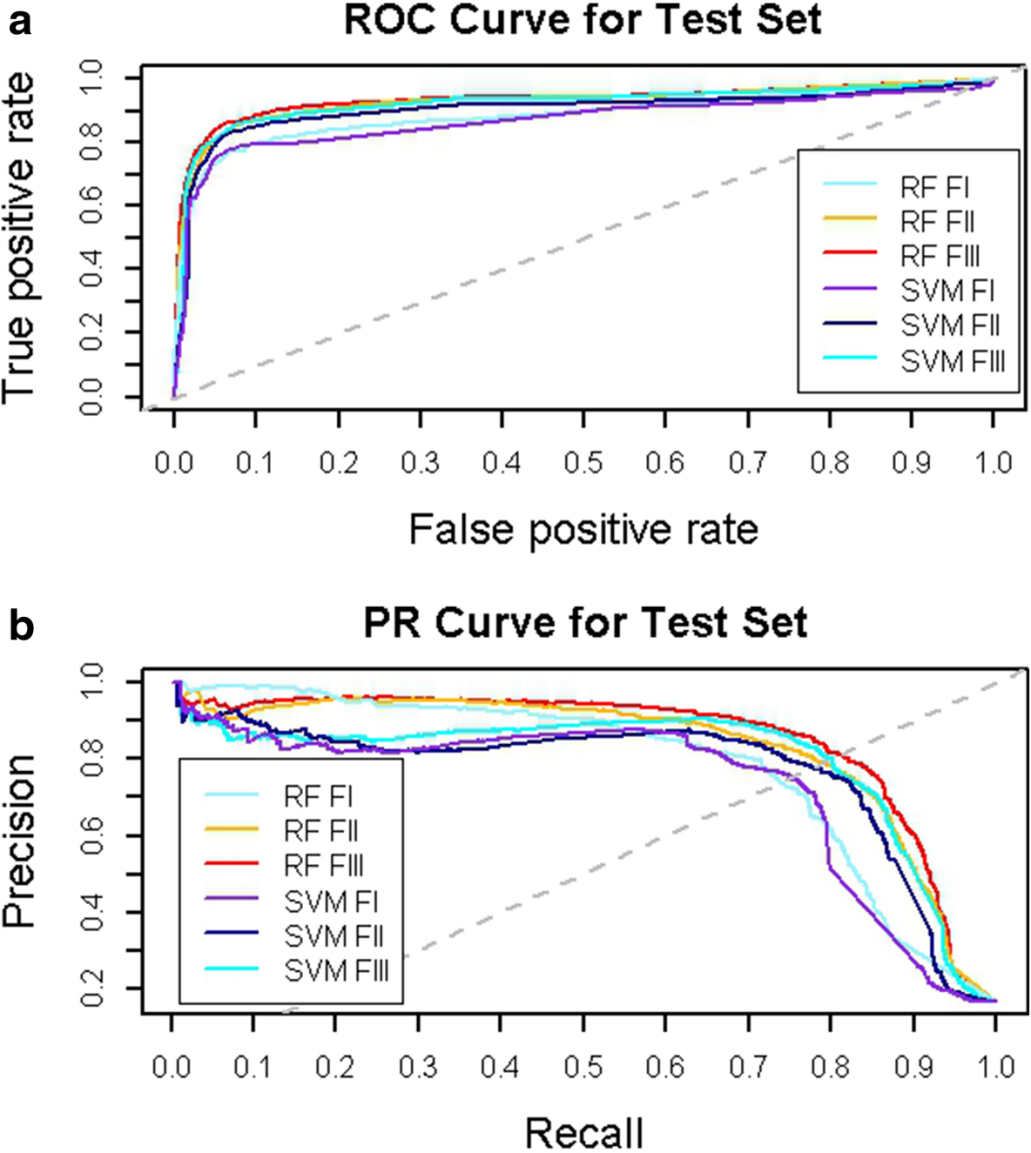
Receiver operating characteristic curves (**a**) and precision/recall curves (**b**) for the six models using two machine learning algorithms to build binary classification models upon three topological feature spaces. RF means Random Forest, SVM means support vector machine, FI means feature set ***I***, FII means feature set ***II***, and FIII means feature set ***III***

The optimal tuning parameters and the statistical results for all the binary classification models are summarized in Table 3. RF outperformed SVM across all three feature sets. Both RF and SVM yielded consistent rankings of the predictive performance for the different feature sets: feature set ***III*** > feature set ***II*** > feature set ***I***. The similarity neighboring links improved the link prediction performance on test set by 5.5 % in RF models, and by around 4.4 % in SVM models. In combination with RW normalization, the predictive performance of RF models was improved by 2 %, and the predictive performance of SVM models were boosted by 3.5 %. The differences in predictive performance were consistently demonstrated by ROC and PR curves as well (see Fig. 2). The ROC space and PR space agreed on the rankings of different feature sets, in terms of predictive performance. We can see that feature set ***III*** dominated both ROC space and PR space for both RF and SVM models, and RF models slightly outperformed SVM models. Since we have imbalanced distributions for positive and negative labels, PR curves can provide better visual representations than ROC curves to identify the difference of predictive performance. As shown in Fig. 2, the ROC curves were closely clustered, and the PR curves for different models were separated to a larger extent. The differences among AUCPRs were larger than the differences among AUCROCs, as well (see Table 4). It is clear that similarity neighboring links are important for link prediction in the semantic network, and RW normalization can boost predictive performance by enriching feature space. It is noteworthy that all the machine learning models performed fairly well on both training and test sets without over-fitting. In addition, both feature set ***II*** and feature set ***III*** produced AUCROCs greater than 0.92, which was produced by SLAP [15]. Hence, meta-path-based topological features have been proven to be valuable for link prediction in complex semantic networks using machine learning models.

**Table 3:** Statistics of binary classification models built upon different feature sets and using different machine learning algorithms

topological feature	Dataset	Random Forest		Support Vector Machine		
		*mtry*	F_1_-score	*C*	*λ*	F_1_-score
Feature set ***I***	Training	12	0.780	8	0.250	0.766
	Test		0.735			0.719
Feature set ***II***	Training	13	0.844	16	0.062	0.810
	Test		0.790			0.763
Feature set ***III***	Training	13	0.859	16	0.016	0.843
	Test		0.810			0.798

**Table 4:** Area under ROC curve (AUCROC) and area under PR curve (AUCPR) of random forest and support vector machine classification models using different feature sets

topological feature	Random Forest		Support Vector Machine	
	AUCROC	AUCPR	AUCROC	AUCPR
Feature set ***I***	0.891	0.772	0.871	0.729
Feature set ***II***	0.927	0.826	0.905	0.768
Feature set ***III***	0.938	0.857	0.922	0.795

**Fig. 3:**
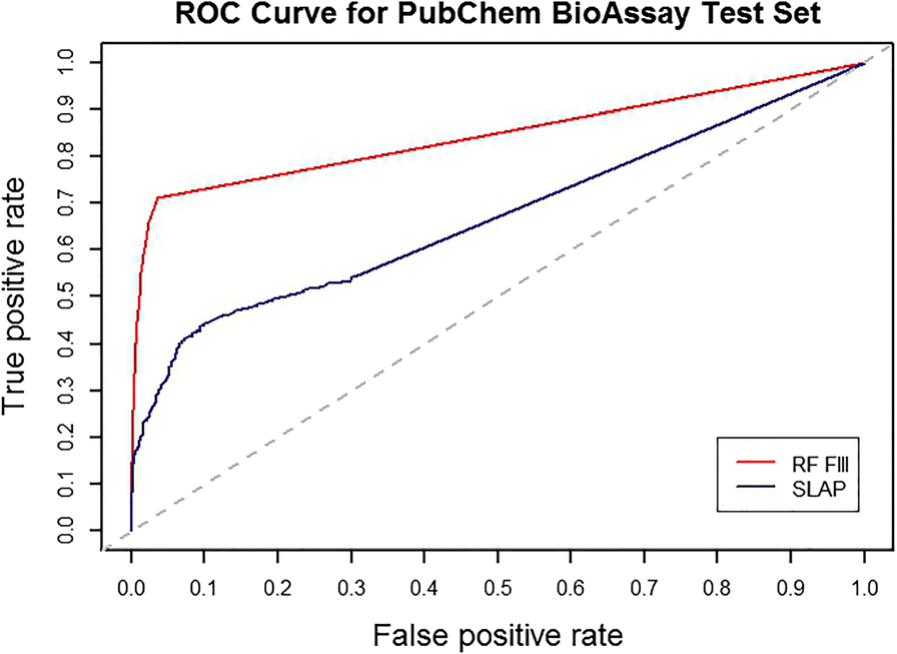
ROC curves for the Random Forest model built upon feature set ***III*** and SLAP. RF means Random Forest and FIII means feature set ***III***

In order to further compare the proposed approached with SLAP, we carried out link predictions using both methods on a large set of unknown links of an evolving semantic network. The labels of those unknown links were derived from experimental evidence deposited in PubChem BioAssay databases after the Chem2Bio2RDF network was constructed. Hence, these positive labels can be viewed as experimental validations when assessing link prediction performance. The proposed framework, using RF to build a binary classification model upon feature set ***III,*** yielded much better BEDROC and AUCROC than SLAP (Table 5). BEDROC is mainly used to compare ranking systems in terms of early recognition [33]. Our approach yielded much better AUC of BEDROC using a default coefficient parameter (α = 20.0) (Table 5). The difference can be seen in Fig. 3 as well.

**Table 5:** Comparing the proposed framework (random forest classification model applied on feature set III) with existing algorithm SLAP using Area under ROC curve (AUCROC) and area under PR curve (AUCPR)

	AUCROC	BEDROC
Feature set ***III***	0.845	0.929
SLAP	0.670	0.672

**Fig. 4:**
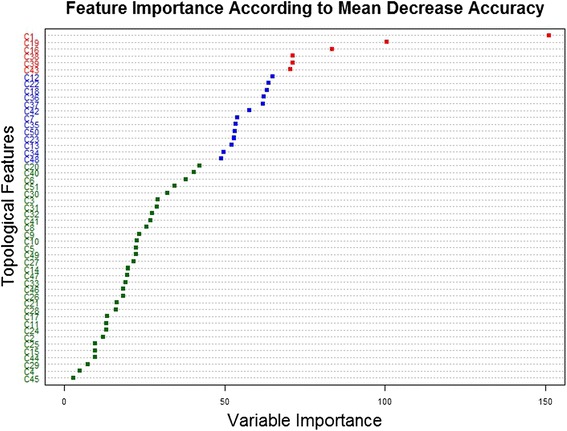
Variable importance for Random Forest model built with feature set ***II***. The color code for feature importance according to mean decrease accuracy: red (>70), blue (>45 and <70), green (<45); the color code for feature importance according to mean decrease Gini index: red (>240), blue (>240 and <100), green (<100)

By applying the intrinsic feature ranking algorithm of the RF on feature set ***II***, we can tell which meta-paths are important for link prediction. Feature importance can be visualized as a dot plot (Fig. 4). Two measures evaluated before and after permutations were used for feature ranking: decrease of classification accuracy and decrease of Gini index. Although two measures do not always agree on which features are important, we still can identify some significantly important meta-paths according to two measures. The top four important meta-paths were C1, C19, C16, and C39, and the network nodes connected by these important meta-paths are compounds, proteins, and GO annotations. It is noteworthy that the top three important meta-paths only contain semantic links between compounds and proteins, and the top two important meta-paths contain similarity neighboring links. Therefore, semantic links between compounds and proteins, including similarity neighboring links and interaction links, played a major role in predicting CPIs.

**Fig. 5:**
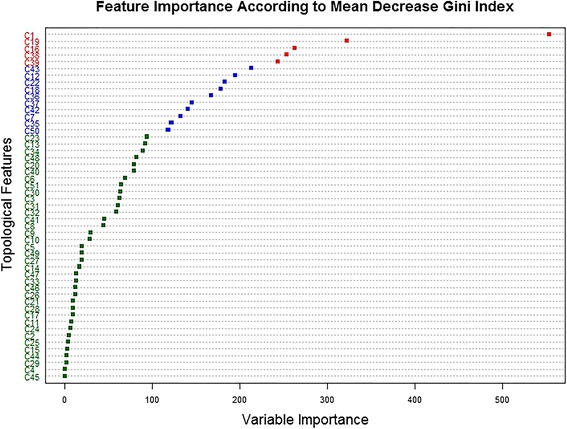
Box plot for the variable importance varying in 1 000 Random Forest models

In contrast to SLAP, that pre-calculates feature importance before making predictions, the proposed framework can evaluate feature importance and build predictive models at the same time. The importance of a given topological feature may vary to some extent when different sets of training data are considered, or when new links are added into the network as a function of time. We carried out an experiment to demonstrate that feature importance may vary significantly when different sets of data are used to build predictive models. We constructed 1,000 RF models using randomly selected training sets with feature set ***II***. Each training set was compiled by 100 positively labeled links from the DrugBank set, and 100 negatively labeled links from the PubChem BioAssay set with experimental bioactivity value greater than 10 μM. The changes of feature importance in different models can be seen in Fig. 5. It is clearly that feature importance varied a lot in different models. Feature C4 has the smallest standard deviation (0.828) and feature C39 has the largest standard deviation (5.537). It is noteworthy that all of the top four importance features in the aforementioned models (C1, C16, C19, and C39) have very large standard deviations. Even though their importance varied a lot in different models, their mean values were well above the average of others; in particular, the mean values of C1 and C39 were much larger than those of other topological features. The predictive performances of those 1,000 RF models tested against a randomly selected set of 50 positive labels and 50 negative labels (not included in any of those 1,000 training sets) varied a lot as well. The highest F_1_-score is 0.937 and the lowest F_1_-score is 0.667. Hence, the selection of training set is also very important to build highly predictive machine learning models.

## Conclusions

The semantic network integrating domain knowledge across chemical and biological space can be leveraged for large-scale data mining. Among the different kinds of semantic links, drug-target connectivity maps have drawn extensive attention, since they are beneficial for drug discovery and development, in particular, drug repositioning and polypharmacology research. In the present work, we have proposed a framework to construct state-of-the-art machine learning models using meta-path-based topological features for link prediction in complex semantic networks. Supervised classification models were shown to be powerful, based on their predictive performance in an independent test set containing links of an evolving network. In addition, the intrinsic feature ranking algorithm embedded in machine learning models can be used to select the most important topological features. Although the proposed framework was only applied to predict DTIs in the present work, it can definitely be used for other purposes, such as to predict associations between drugs and adverse side effects, as well as associations between proteins and diseases. In the future, we want to study how to select the most relevant training set for a given prediction task, and how much training set selection can improve predictive performance.

### Availability of Data and Materials

The data sets supporting the results of this article are included within the article and its additional files (Additional files 1, 2, 3, 4 and 5).

## Additional files

Additional file 1: (Cpluspluscodes.zip): it contains the C++ codes to generate adjacent matrix and compute commuting matrix. (ZIP 9294 kb) Additional file 2: (Rcodes.zip): it contains the R codes to apply machine learning algorithms on the commuting matrix feature space to construct binary classification model. (ZIP 48844 kb) Additional file 3: (semantic_network_dataset.zip): it contains the semantic links of the augmented Chem2Bio2RDF graph and the labels for internal test set, which has been blinded out from training set. (ZIP 26931 kb) Additional file 4: (metapath_commuting_matrix.zip): it contains the calculated commuting matrix for internal and external validating. The internal test set was used by SLAP in the original publication; the external test set was prepared in the present study and intended to examine the ability to predict evolving network topologies. (ZIP 27340 kb) Additional file 5: (evolving_network_prediction.zip): it contains the predicted results using SLAP and presented meta-path-based semantic network analysis framework. (ZIP 4712 kb)

## Abbreviations

FN: false negative
FP: false positive
MCC: Mathews correlation coefficient
PC: path count
RF: random forest
RW: random walk
SLAP: semantic link association prediction
TN: true negative
TP: true positive
